# Postpartum cardio-obstetrics rehabilitation program for women after hypertensive pregnancy: A single-arm proof-of-concept study

**DOI:** 10.1038/s41440-026-02556-1

**Published:** 2026-01-28

**Authors:** Karan Pongpanit, Garvee Patel, Lishana Sellan, Léna Nguyen, Michelle Jewett, Gregory Moullec, Simone Marques Gomes, Joelle Labonté, Cindy Kwan, Sonia Gagnon, Isabelle Vachon, Tania Janaudis-Ferreira, Marc Roig, Mariane Bertagnolli

**Affiliations:** 1 School of Physical and Occupational Therapy, Faculty of Medicine and Health Sciences, McGill University, Montreal, Quebec Canada; 2 Department of Physical Therapy, Faculty of Allied Health Sciences, Thammasat University, Pathum Thani, Thailand; 3 Centre Intégré Universitaire en Santé et Services Sociaux (CIUSSS) du Nord-de-l’Île-de-Montréal, Montreal, Quebec Canada; 4 Cardiovascular Health Across the Lifespan Program, Research Institute of the McGill University Health Centre, Montreal, Quebec Canada; 5 Hôpital Maisonneuve-Rosemont, CIUSSS de l’Est-de-l’Île-de-Montréal, Montreal, Quebec Canada; 6 Respiratory Epidemiology and Clinical Research Unit, Centre for Outcomes Research and Evaluation, Research Institute of the McGill University Health Centre, Montreal, Quebec Canada; 7 Memory and Motor Rehabilitation Laboratory (Memory-Lab), Center for Interdisciplinary Research in Rehabilitation of Greater Montreal (CRIR), Montreal, Quebec Canada

**Keywords:** Rehabilitation, Hypertensive pregnancy, Postpartum, Implemental hypertension, Feasibility

## Abstract

Hypertensive disorders during pregnancy increase the risk of long-term cardiovascular disease in postpartum women. Exercise-based rehabilitation may help manage blood pressure (BP) and improve physical activity levels in this population, but supporting evidence remains limited. This pre-post single-arm proof-of-concept study aimed to assess the feasibility of a 4-week cardio-obstetrics rehabilitation program for women following hypertensive pregnancy. Women 3–6 months postpartum with a history of gestational hypertension or pre-eclampsia were recruited. The intervention combined exercise and educational components delivered through in-person, live virtual, and independent sessions. Feasibility was evaluated through recruitment, retention, adherence, acceptability, and safety. Outcomes included BP, six-minute walk distance, body weight and BMI, physical activity levels, health-related quality of life, and depressive symptoms. Six of 20 screened participants (30% recruitment) completed the intervention (100% retention). Overall adherence to scheduled sessions was 71%. All participants expressed high satisfaction, and no adverse events were reported. Descriptive analysis indicated improvements across all measured outcomes after the intervention. A cardio-obstetrics rehabilitation program for postpartum women after hypertensive pregnancy is feasible. Improvements in cardiovascular, anthropometric, behavioral, and psychosocial outcomes suggest potential efficacy and support further investigation.

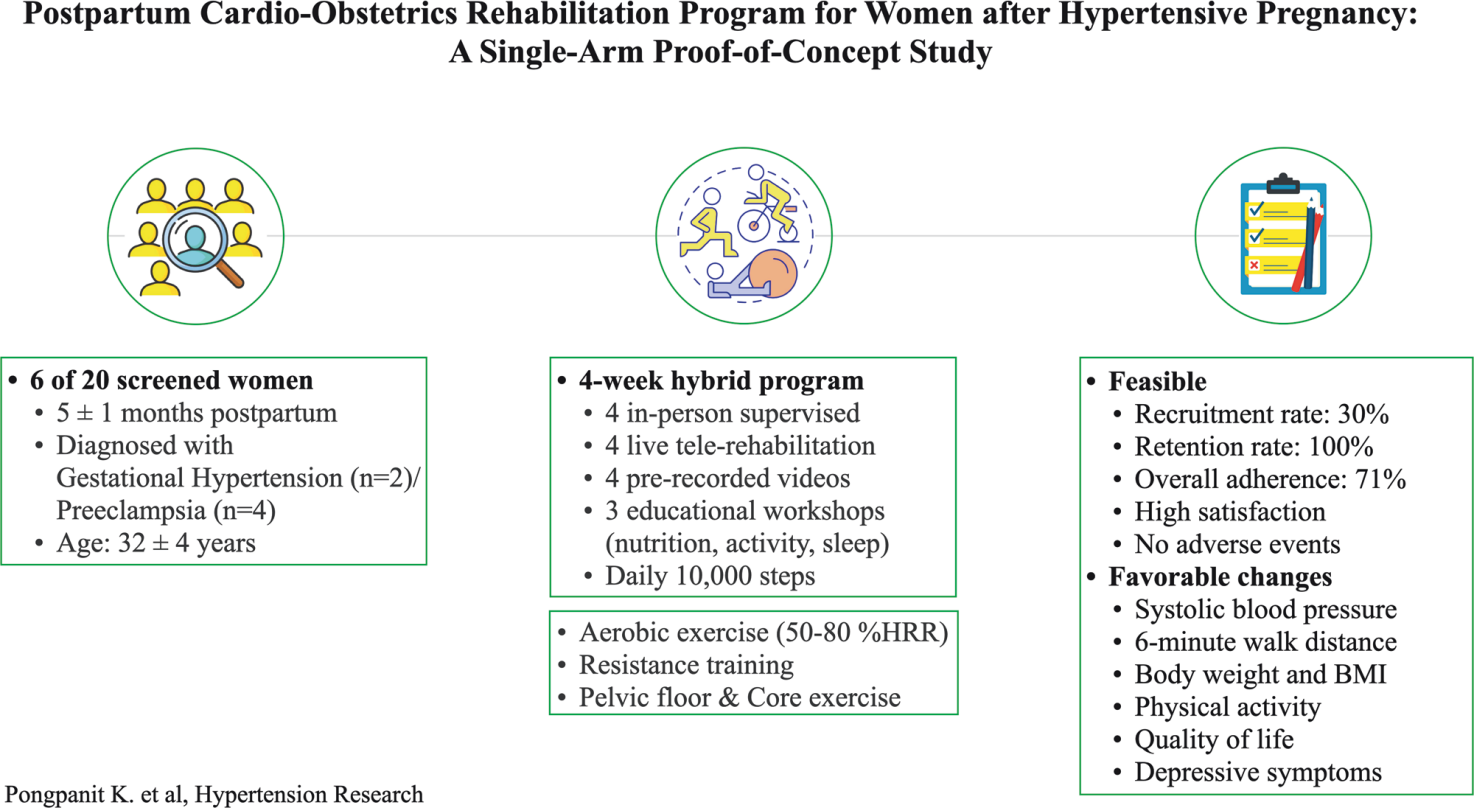

## Introduction

Hypertensive disorders of pregnancy (HDP) are major contributors to maternal morbidity and mortality globally [1]. Beyond their acute complications during gestation and delivery, HDP impose a lasting burden on cardiovascular health, with growing evidence linking them to an elevated risk of chronic hypertension, heart failure, cerebrovascular disease, and cardiomyopathy later in life [2, 3]. More specifically, HDP are frequently associated with blood pressure (BP) instability during the early postpartum period, with persistently elevated BP beyond 12 weeks potentially indicating the progression to chronic hypertension [4, 5]. As such, the postpartum period has increasingly been recognized not only as a time of recovery, but also as a crucial window for initiating long-term cardiovascular disease prevention strategies.

Despite this recognition, clinical care following HDP remains limited, with few structured non-pharmacological interventions targeting the cardiovascular risks associated with these conditions [6]. Recent Canadian guidelines recommend the adoption of lifestyle modifications, including physical activity and exercise, for the first year following childbirth. Specifically, at least 120 min per week of moderate-to-vigorous intensity aerobic activity is advised to promote cardiometabolic health [7]. However, evidence on the most effective ways to implement such interventions in postpartum women, particularly those recovering from hypertensive pregnancy complications, remains inconclusive.

A recent systematic review and meta-analysis from our research group revealed that, of the postpartum exercise interventions specifically targeting maternal vascular health and BP published to date, two involved postpartum women [8], with only one study by Riemer et al. [9] explicitly focusing on women with a history of preeclampsia (PE). This study demonstrated that a 24-week postpartum cardiovascular lifestyle intervention, integrating supervised exercise and dietary counseling, was well tolerated and led to significant vascular improvements compared to a reference group.

HDP are physiologically associated with some persistent endothelial dysfunction, increased arterial stiffness, heightened systemic inflammation, and metabolic dysregulation, all of which may continue well into the postpartum period and contribute to long-term cardiovascular remodeling [10]. Structured physical activity and exercise have been shown to mitigate many of these pathophysiological changes in both general and cardiac-specific populations [11–13], suggesting potential benefits for women recovering from HDP. However, unique challenges, such as physical exhaustion, emotional challenges, competing demands on time, limited support, and low motivation, often hinder engagement in this population [14]. Rehabilitation programs tailored to this cohort must, therefore, be flexible, supportive, and feasible within the complex realities encountered in the period following delivery.

In response to these clinical and scientific gaps, this study aimed to evaluate the feasibility of a postpartum cardio-obstetrics rehabilitation program for postpartum women with a recent history of HDP. Specifically, we sought to: (i) assess the recruitment, retention, adherence, acceptability, and safety of a 4-week program combining exercise and educational components; and (ii) estimate the extent to which the designed intervention affects maternal BP, functional capacity, anthropometrics, physical activity levels, health-related quality of life, and depressive symptoms.

## Methods

### Study design

This study was designed as a pre-post single-arm proof-of-concept study. Reporting of the findings follows the CONSORT extension for pilot and feasibility trials [15]. The study was approved by the Centre Intégré Universitaire de Santé et de Services Sociaux du Nord-de-l'Île-de-Montréal (CIUSSS-NÎM) and all participants provided informed consent. Given the exploratory nature of this study, the sample size was based on feasibility, and, in line with the ORBIT model for behavioral treatment development, a control group was not required at this stage [16].

### Participants

Participants were recruited between May and July 2022 from the Clinique de Grossesse à Risque at the Mother-Child Unit of the Hôpital du Sacré-Cœur de Montréal (HSCM), CIUSSS-NÎM. Eligible individuals were identified by their obstetricians, who referred those meeting the study criteria to the research team. Potential participants were then contacted by telephone, provided with detailed information about the study procedures and associated risks.

Women were eligible for inclusion if they were 18 years of age or older, between three and six months postpartum, had been diagnosed with new-onset hypertension (≥140/90 mmHg) after 20 weeks of gestation, with either the absence (gestational hypertension, GH) or presence (PE) of proteinuria or maternal organ involvement, and were not taking antihypertensive medication at the time of enrollment. Participants were also required to be able to communicate in English or French and have access to a computer or mobile device with internet connectivity. The selected postpartum timeframe aligns with typical recovery trajectories and reflects an expected period of physical and psychological readiness for re-engaging in structured physical activity, which often occurs between 8 and 12 weeks following delivery [17].

Participants were excluded if they had a diagnosis of chronic hypertension, were currently using antihypertensive medication, had major contraindications to physical activity (e.g., cardiac conditions, neurological or musculoskeletal disorders), or exhibited significant cognitive impairment or mental health conditions that could affect informed consent and study participation.

### Intervention

The intervention comprised a structured, four-week cardio-obstetrics rehabilitation program integrating both exercise and educational components. The four-week duration was selected based on existing evidence suggesting that this timeframe provides sufficient opportunity for behavioral change [18] and cardiovascular adaptation to exercise [19], while remaining practical and feasible for postpartum women. Participants were expected to complete a total of 15 sessions over the intervention period, including four in-person supervised exercise sessions, four live telerehabilitation sessions, four independently completed sessions using pre-recorded videos, and three educational workshops delivered either online or in pre-recorded format. A hybrid format, combining both educational and physical components, was used to evaluate the acceptability, preference, and adherence to these different delivery methods.

Each exercise session followed a standardized structure, involving 5 min of warming up, 30 min of aerobic training, 10 min of resistance exercises, 5 min of core strengthening, 5 min of pelvic floor rehabilitation, and 5 min of stretching and relaxation. Exercise difficulty and intensity were progressively increased throughout the program. During the first week, aerobic training was performed at 50–60% of estimated heart rate reserve, increasing to 60–70% in weeks two and three, and reaching 70–80% by week four. Resistance exercise was prescribed at an intensity of 0–60% of one-repetition maximum with two to four sets of 8–12 repetitions per exercise and was adjusted based on participants’ ability and tolerance.

In-person sessions took place at the Centre Jean-Jacques-Gauthier (CJJG), CIUSSS-NÎM, and were supervised by a trained member of the research team. Aerobic training was performed using either a treadmill or stationary bike, with BP, heart rate, oxygen saturation, and perceived exertion recorded before and after each session to ensure safety and monitor physiological response. Participants were also instructed to complete one weekly telerehabilitation session delivered via Zoom under the supervision of a team member, as well as one independent session using a pre-recorded video accessed through a private YouTube channel. For both formats, they were asked to engage in 30 to 60 min of walking or aerobic activity on a self-selected day, guided by individualized targets using step counts or heart rate ranges. To facilitate self-monitoring of daily steps and exercise heart rate range and support adherence, participants were provided with a Fitbit Inspire 2 (Fitbit Inc., San Francisco, CA, USA) and a suggested daily step goal of 10,000 steps [20].

Regarding educational workshops, participants attended sessions delivered by members of the CJJG team that addressed key postpartum health topics, including nutrition, physical activity, and sleep hygiene. At the initial visit, they also received a printed leaflet outlining evidence-based physical activity recommendations for the postpartum period.

### Outcome measures

Participants underwent assessments at two time points: the initial baseline visit, which took place between three to six months postpartum, and a follow-up visit after completing the four-week rehabilitation program. During the initial assessment, sociodemographic data, medical and perinatal history, and behavioral information were collected through questionnaires and review of the medical chart.

#### Implementation feasibility

Feasibility outcomes included recruitment, retention, adherence, acceptability, and safety. Adherence was tracked by documenting attendance at in-person and virtual sessions and reviewing completed logbooks for pre-recorded sessions. Acceptability was assessed through post-intervention feedback questionnaires. Safety was monitored by recording BP, heart rate, oxygen saturation, and perceived exertion during supervised sessions. Adverse events were defined as any unintended harmful experiences participants encountered during the intervention, which may include, but are not limited to, injuries, dizziness, fainting, severe discomfort, or other related issues [21]. These events were documented through session records and participant logs.

Quantitative physiological responses included resting BP, functional capacity, anthropometrics, and physical activity levels. Systolic and diastolic BP were measured after 10 min of seated rest using a validated automated sphygmomanometer (Omron Healthcare Co. Ltd, Kyoto, Japan). Three readings were taken at two-minute intervals, and the average of the second and third readings was used for analysis. Functional capacity was evaluated using the six-minute walk test (6MWT) following American Thoracic Society guidelines [22]. With subjects barefoot and fasted, height was measured using a wall-mounted stadiometer (Seca North America, Chino, CA, USA) and weight was measured on an electronic standing scale (Detecto, Webb City, MO, USA). Body mass index (BMI) was then calculated as weight in kilograms divided by the square of height in meters. Objective physical activity data, including step count and activity duration, were collected using a Fitbit Inspire 2, which participants were instructed to wear throughout the four-week program.

#### Self-reported health outcomes

Participants’ physical activity levels were also self-reported using the Pregnancy Physical Activity Questionnaire (PPAQ), while perceived functional capacity was assessed with the Duke Activity Status Index (DASI). Quality of life was measured using the 36-Item Short Form Health Survey (SF-36), and psychological well-being was evaluated using the Edinburgh Postnatal Depression Scale (EPDS) and the Patient Health Questionnaire-9 (PHQ-9).

Post-intervention feedback was collected through a custom-designed questionnaire administered via REDCap, which included both closed-ended items (5-point Likert scale) and open-ended questions. The Exercise Benefits and Barriers Scale (EBBS) was used to assess attitudes toward exercise, while perceptions of the rehabilitation process were captured using the Cardiac Rehabilitation Facilitators (CRFS) and Barriers Scale (CRBS).

### Statistical analysis

Descriptive statistics were used to characterize participant demographics and outcomes. Continuous variables are reported as means with standard deviations or medians with interquartile ranges according to distribution. Changes between baseline and follow-up were reported using mean or median differences alongside effect sizes (ES) calculated with Hedges’ g, interpreted as small (0.2), medium (0.5), or large (0.8) [23]. Minimal clinically important differences (MCIDs) were employed to contextualize the clinical relevance of observed changes in physical outcomes, specifically 2 mmHg for BP [24, 25], and 30 meters for the six-minute walk distance (6MWD) [26].

## Results

**Fig. 1:**
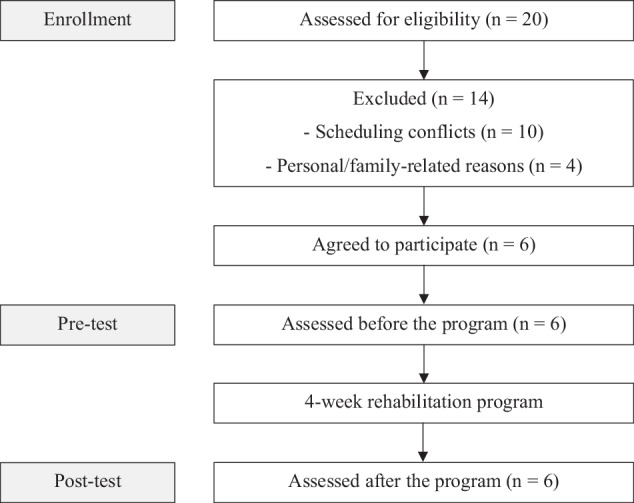
CONSORT flow diagram

Of the 20 women screened, six (30% recruitment) met the inclusion criteria and were enrolled in the study. All participants completed the intervention, yielding a 100% retention rate (Fig. 1). Baseline characteristics of the participants are shown in Table 1.

**Table 1:** Participant characteristics

	Participants included (*n* = 6)
**Maternal demographics**	
Maternal age (years)	32.33 ± 4.37
Postpartum period (months)	5.50 ± 0.55
Immigrant [n (%)]	6 (100)
Racial and ethnic [n (%)]	
Black	4 (66.67)
Asian	1 (16.67)
White	1 (16.67)
Family status [n (%)]	
Married or domestic relationship	6 (100)
Level of education [n (%)]	
University, college, or equivalent	6 (100)
Use of drugs or alcohol [n (%)]	
No	5 (83.33)
**Maternal anthropometrics and blood pressure**	
First-trimester	
Body weight (kg)	87.27 ± 26.95
Body mass index (kg/m^2^)	29.91 ± 5.90
Systolic blood pressure, (mmHg)	118.33 ± 9.97
Diastolic blood pressure, (mmHg)	75.17 ± 7.55
At birth	
Body weight (kg)	98.18 ± 28.38
Body mass index (kg/m^2^)	34.50 ± 6.44
Systolic blood pressure, (mmHg)	150.67 ± 12.68
Diastolic blood pressure, (mmHg)	92.00 ± 4.98
**Pregnancy complications**	
Hypertensive disorders of pregnancy [n (%)]	
Gestational hypertension (GH)	2 (33.33)
Preeclampsia (PE)^*^	4 (66.67)
Other pregnancy complications [n (%)]	
Gestational diabetes	3 (50.00)
Obesity	4 (66.67)
Anemia	4 (66.67)
Hypothyroidism	2 (33.33)
Chorioamnionitis	1 (1.67)
**Delivery characteristics**	
Mode of delivery [n (%)]	
Vaginal	3 (50)
Caesarean	3 (50)
Gestational age (weeks)	38.33 ± 1.51
**Infant outcomes**	
Infant sex assigned [n (%)]	
Female	4 (66.67)
Infant birth weight (g)	3410.00 ± 470.41
Fetal distress [n (%)]	
APGAR at 1 min, < 7	3 (50.00)
APGAR at 5 min, < 7	2 (33.33)
APGAR at 10 min, < 7	0 (0.00)
Breastfeeding [n (%)]	
Exclusive	1 (16.67)

Values are presented as mean ± SD, if not mentioned otherwise

^*^Two participants initially diagnosed with GH later progressed to PE

### Adherence

Overall adherence to the rehabilitation program was 71%, with individual adherence ranging from 48% to 85%. The highest adherence was observed for pre-recorded exercise videos (79%; range 25%–100%), whereas the lowest was noted for telerehabilitation sessions (67%; range 25%–100%) and educational workshops (67%; range 33%–100%). Adherence to in-person sessions averaged 71% (range 50%–100%). The most frequently reported reasons for missed sessions were time constraints, primarily related to caregiving responsibilities such as caring to a sick child, difficulty arranging childcare, or managing other household demands.

### Acceptability

All participants (100%) agreed that the program was beneficial for their health, provided useful exercises they intended to continue, and that they were satisfied with the overall delivery format. Five participants (83.33%) found the program engaging and reported that the session formats supported their physical activity and sustained engagement. All participants (100%) found each session format easy to follow, although two (33.33%) described the formats as physically demanding and time-consuming (Supplementary Table 1).

Open-ended feedback highlighted a desire for greater scheduling flexibility, including the option of weekend sessions. Participants valued group-based formats for enhancing motivation and fostering community, with in-person and live virtual sessions generally preferred over pre-recorded content. Suggestions included extending the program duration to allow for rescheduling missed sessions, as well as expanding educational topics to cover stress management and postpartum well-being. While most participants were comfortable enrolling independently, one noted that referrals from healthcare providers might further improve motivation. Seasonal timing also emerged as a factor, with a preference for summer to avoid weather-related barriers. All participants reported improvements in physical and mental health, alongside positive lifestyle changes.

### Adverse events

Throughout the four-week intervention, no adverse events were reported in any part of the program. Participants’ safety and well-being were consistently monitored, and no complications or negative effects were observed.

### Physical and behavioral changes after the rehabilitation program

Physical outcome changes following the rehabilitation program are presented in Table 2 and Supplementary Fig. 1. A tendency toward improvement was observed in BP, functional capacity, and anthropometrics, with positive changes found in systolic BP (ES = 0.696), 6MWD (ES = 0.278), body weight (ES = 0.763), and BMI (ES = 0.550). When compared to established MCIDs [24–26], 4 participants demonstrated either improvement or maintenance in systolic BP, 2 in diastolic BP, and 3 in 6MWD.

**Table 2:** Change in physical outcomes after the 4-week postpartum cardio-obstetric rehabilitation program

		Baseline	Post 4 weeks	Mean change	Effect size
Systolic blood pressure (mmHg)	mean ± SD	124.83 ± 12.80	119.83 ± 8.57	-5.00 ± 7.18	0.696
	median (min-max)	123.50 (112.00-146.00)	120.50 (107.00-130.00)	-4.00 (-19.00-1.00)	
Diastolic blood pressure (mmHg)	mean ± SD	88.33 ± 10.97	89.67 ± 6.15	1.33 ± 10.65	0.125
	median (min-max)	89.50 (76.00-104.00)	87.50 (84.00-101.00)	6.00 (-16.00-11.00)	
Six-minute walk distance (m)	mean ± SD	524.16 ± 57.94	536.17 ± 34.92	12.01 ± 43.19	0.278
	median (min-max)	513.58 (465.00-627.00)	543.30 (475.60-573.73)	10.60 (-65.50-64.10)	
Body weight (kg)	mean ± SD	89.45 ± 26.12	87.97 ± 25.40	-1.48 ± 1.94	0.763
	median (min-max)	91.60 (57.80-130.40)	(56.00-125.50)	-1.45 (-4.90-0.80)	
Body mass index (kg/m^2^)	mean ± SD	31.50 ± 5.68	30.83 ± 5.85	-0.54 ± 0.75	0.550
	median (min-max)	31.50 (24.00-40.00)	31.00 (22.00-38.00)	-0.46 (-1.49-0.51)	

Effect size was calculated with Hedges’ g

Table 3 shows the observed behavioral changes. Self-reported physical activity levels, measured using the PPAQ, showed domain-specific changes post-intervention. Increases in moderate- and vigorous-intensity activities, as well as total activity levels, were noted, with mean changes of 21.46 metabolic equivalent hours per week (MET-h/week) (ES = 1.309), 2.44 MET-h/week (ES = 0.815), and 7.69 MET-h/week (ES = 0.268), respectively. Conversely, light-intensity activity decreased by a mean of 32.32 MET-h/week (ES = 0.858), while sedentary activity remained stable (mean change = 0.73 MET-h/week, ES = 0.047). Objective physical activity data further supported these behavioral improvements, revealing increases in daily step count (531.20 steps, ES = 2.087), light activity time (30.22 min, ES = 1.281), and moderate activity time (6.94 min, ES = 0.770). Sedentary time was reduced by 171.05 min (ES = 0.961), and vigorous activity time decreased by 1.65 min (ES = 0.137).

**Table 3:** Behavioral changes after the 4-week postpartum cardio-obstetric rehabilitation program

		Baseline	Post 4 weeks	Mean change	Effect size
*PPAQ* (MET-h/week)					
Total activity	mean ± SD	258.88 ± 77.10	266.56 ± 92.83	7.69 ± 28.65	0.268
	median (min-max)	258.72 (140.53-341.38)	252.33 (160.20-392.60)	14.62 (-27.63-52.22)	
Sedentary activity	mean ± SD	46.35 ± 33.03	47.08 ± 34.35	0.73 ± 15.48	0.047
	median (min-max)	36.40 (21.53-109.73)	37.10 (19.25-114.98)	-6.30 (-20.20-14.87)	
Light-intensity activity	mean ± SD	136.77 ± 61.52	104.45 ± 30.20	-32.32 ± 37.67	0.858
	median (min-max)	146.48 (51.98-208.60)	103.61 (67.03-143.50)	-37.98 (-77.35-15.05)	
Moderate-intensity activity	mean ± SD	80.62 ± 45.48	102.08 ± 38.69	21.46 ± 16.40	1.309
	median (min-max)	78.55 (14.20-151.98)	109.88 (41.95-157.73)	26.25 (5.75-48.82)	
Vigorous-intensity activity	mean ± SD	2.84 ± 4.21	5.28 ± 5.10	2.44 ± 2.99	0.815
	median (min-max)	0.82 (0.00-10.50)	3.38 (1.63-15.00)	3.38 (-1.50-6.63)	
*Fitbit*					
Number of steps (steps)	mean ± SD	6254.00 ± 2408.98	6785.20 ± 2208.62	531.20 ± 254.52	2.087
	median (min-max)	5296.00 (4683.50-8303.50)	5793.00 (5406.50-8660.00)	514.00 (199.00-915.00)	
Sedentary time (min)	mean ± SD	938.48 ± 244.18	767.42 ± 120.32	-171.05 ± 177.95	0.961
	median (min-max)	872.25 (772.17-1137.90)	745.90 (656.61-889.00)	-126.35 (-419.00-1.00)	
Light activity time (min)	mean ± SD	179.99 ± 82.81	210.21 ± 77.74	30.22 ± 23.60	1.281
	median (min-max)	158.50 (110.25-260.48)	218.88 (143.05-273.05)	24.52 (0.60-62.38)	
Moderate activity time (min)	mean ± SD	14.08 ± 13.23	21.02 ± 20.12	6.94 ± 9.00	0.770
	median (min-max)	10.50 (5.92-24.04)	13.70 (6.93-38.77)	2.95 (-1.40-17.71)	
Vigorous activity time (min)	mean ± SD	19.51 ± 20.66	17.86 ± 13.60	-1.65 ± 12.07	0.137
	median (min-max)	10.25 (5.48-38.17)	11.10 (6.49-32.61)	0.85 (-21.62-10.50)	
*DASI*					
Total score	mean ± SD	42.20 ± 12.77	45.74 ± 13.68	3.54 ± 4.88	0.610
	median (min-max)	44.70 (26.70-58.20)	46.58 (32.20-58.20)	3.50 (-4.25-8.00)	
Estimated VO_2_ peak (mL/kg/min)	mean ± SD	27.75 ± 5.49	29.27 ± 5.88	1.52 ± 2.10	0.610
	median (min-max)	28.83 (21.08-34.63)	29.63 (23.45-34.63)	1.51 (-1.83-3.44)	
Estimated METs (MET-h/week)	mean ± SD	7.93 ± 1.57	8.36 ± 1.68	0.44 ± 0.60	0.610
	median (min-max)	8.24 (6.02-9.89)	8.47 (6.70-9.89)	0.43 (-0.52-0.98)	
*SF-36 survey*					
Physical functioning	mean ± SD	70.83 ± 22.89	79.17 ± 27.28	8.33 ± 16.02	0.516
	median (min-max)	70.00 (35.00-95.00)	92.50 (40.00-100.00)	5.00 (-20.00-25.00)	
Role-Physical	mean ± SD	54.17 ± 40.05	91.67 ± 12.91	37.50 ± 34.46	0.752
	median (min-max)	50.00 (0.00-100.00)	100.00 (75.00-100.00)	25.00 (0.00-75.00)	
Bodily pain	mean ± SD	60.42 ± 21.82	74.58 ± 32.73	14.16 ± 37.84	0.374
	median (min-max)	51.25 (45.00-100.00)	90.00 (22.50-100.00)	32.50 (-47.50-55.00)	
General health	mean ± SD	58.50 ± 14.47	70.00 ± 23.45	11.50 ± 17.99	0.639
	median (min-max)	55.00 (41.00-80.00)	67.50 (35.00-100.00)	10.00 (-15.00-40.00)	
Vitality	mean ± SD	51.67 ± 17.51	66.67 ± 24.01	15.00 ± 30.82	0.487
	median (min-max)	50.00 (30.00-80.00)	62.50 (30.00-100.00)	20.00 (-25.00-55.00)	
Social functioning	mean ± SD	77.08 ± 12.29	87.50 ± 20.92	10.42 ± 16.61	0.577
	median (min-max)	81.25 (62.50-87.50)	100.00 (50.00-100.00)	12.50 (-12.50-12.50)	
Role-Emotional	mean ± SD	72.22 ± 44.31	94.45 ± 13.61	22.22 ± 40.37	0.548
	median (min-max)	100.00 (0.00-100.00)	100.00 (66.67-100.00)	0.00 (0.00-100.00)	
Emotional Well-Being	mean ± SD	67.33 ± 18.83	82.00 ± 19.39	14.67 ± 28.02	0.523
	median (min-max)	68.00 (44.00-88.00)	88.00 (48.00-100.00)	0.00 (-8.00-56.00)	
*PHQ-9*					
Total score	mean ± SD	5.00 ± 4.10	3.67 ± 4.41	-1.33 ± 4.13	0.323
	median (min-max)	5.00 (0.00-9.00)	3.00 (0.00-12.00)	-2.00 (-6.00-4.00)	
*EPDS*					
Total score	mean ± SD	6.67 ± 2.58	5.17 ± 3.76	-1.50 ± 3.45	0.435
	median (min-max)	6.50 (3.00-11.00)	5.00 (0.00-11.00)	-1.00 (-4.00-4.00)	
Possible depression (10-12)	n (%)	1 (16.67)	1 (16.67)		
Probable depression (≥13)	n (%)	0 (0.00)	0 (0.00)		

Effect size was calculated with Hedges’ g. *DASI* Duke Activity Status Index, *EPDS* Edinburgh Postnatal Depression Scale, *PHQ-9* Patient Health Questionnaire-9; *PPAQ* Pregnancy Physical Activity Questionnaire

Regarding the DASI, the total score increased by a mean of 3.54 points (ES = 0.610), estimated maximal oxygen uptake rose by 1.52 mL/kg/min (ES = 0.610), and estimated metabolic equivalent of task showed a slight increase of 0.44 MET-h/week (ES = 0.610). Similarly, the SF-36 demonstrated improvements across all domains, with the most notable increases in role-physical (37.50 points, ES = 0.752), physical functioning (8.33 points, ES = 0.516), and general health (11.50 points, ES = 0.639). Depressive symptoms exhibited reductions, with the PHQ-9 decreasing by 1.33 points (ES = 0.323) and the EPDS by 1.50 points (ES = 0.435).

### Perceived benefits and barriers to physical activity

**Fig. 2:**
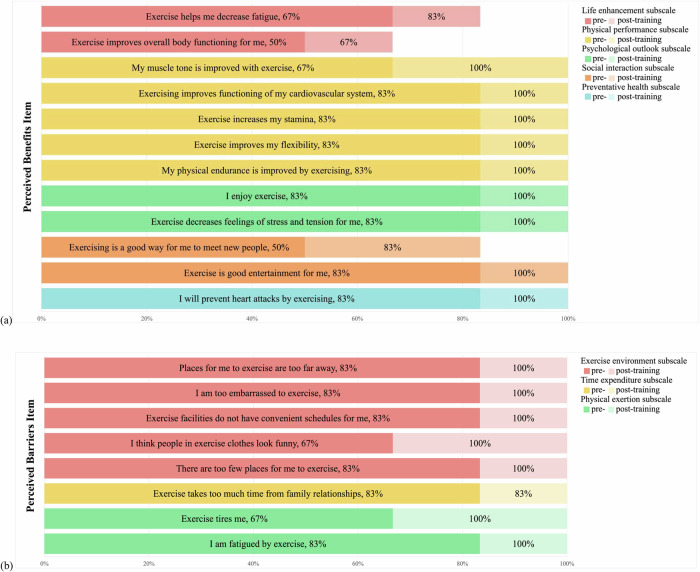
Distribution of perceived exercise benefits **(a)** and barriers **(b)** at baseline and post-intervention, based on the Exercise Benefits/Barriers Scale (EBBS)

Figure 2a and Supplementary Table 2 illustrate responses on exercise benefits from the EBBS, collected at baseline and after the training program. Participants reported increased agreement that exercise helps reduce fatigue (from 67% to 83%) and improves overall body functioning (from 50% to 67%). Physical benefits were more widely endorsed post-intervention, with all participants (100%) reporting that exercise improved muscle tone, cardiovascular health, stamina, flexibility, and physical endurance. Social aspects of exercise were viewed more favorably, with more participants recognizing it as an opportunity to meet new people (from 50% to 83%) and a form of entertainment (from 83% to 100%). Beliefs in exercise’s preventative health benefits also strengthened, particularly regarding its role in preventing heart attacks (from 83% to 100%).

In terms of barriers to exercise, several concerns were more frequently reported after the intervention (Fig. 2b and Supplementary Table 2). Agreement increased across all items related to logistical and environmental challenges, with 100% of participants expressing concerns about distant exercise locations, inconvenient schedules, limited facilities, feeling embarrassed while exercising, and discomfort with others’ appearance in exercise clothing. Moreover, a greater proportion of participants indicated that exercise interfered with family relationships (from 83% to 100%). Perceptions of physical difficulty also rose, with more participants reporting that exercise was tiring (from 67% to 100%) and fatiguing (from 83% to 100%).

### Cardiac rehabilitation-specific facilitators and barriers

**Fig. 3:**
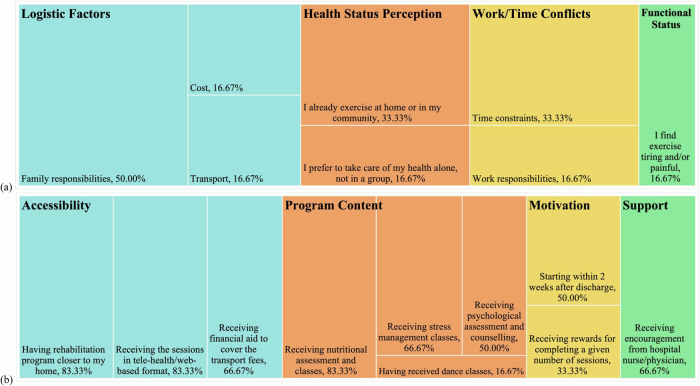
Tree charts of cardiac rehabilitation barriers **(a)** and facilitators **(b)**, based on the Cardiac Rehabilitation Barriers Scale (CRBS) and the Cardiac Rehabilitation Facilitators Scale (CRFS), respectively

Figure 3 and Supplementary Table 3 display participant responses to the CRBS and CRFS. Family responsibilities were identified as the most frequently reported barrier to participation (50.00%), followed by time constraints and the perception that sufficient physical activity could be achieved through home- or community-based exercise (both 33.33%). Participants most frequently identified the proximity of the program to their homes, the availability of tele-rehabilitation options, and the inclusion of nutritional education classes as key facilitators (all 83.33%). Additional commonly cited facilitators included transportation assistance, stress management sessions, and support from healthcare providers (all 66.67%).

## Discussion

This proof-of-concept study provided evidence supporting the feasibility, acceptability, safety, and potential benefits of a postpartum cardio-obstetrics rehabilitation program for women with a history of hypertensive pregnancy. All six participants successfully completed the four-week intervention, demonstrating high engagement and retention. Preliminary findings suggest the program promoted cardiovascular health, improved functional capacity, modulated body weight and BMI, increased physical activity levels, enhanced overall quality of life, and reduced depressive symptoms, while maintaining a safe environment throughout the training.

### Feasibility and participant engagement

The high adherence rate observed in this study indicates strong engagement with the intervention, which supports its feasibility and acceptability among postpartum women. This is particularly notable given common barriers such as physical fatigue, psychological distress, time constraints, limited support, lack of knowledge about healthy behaviors, and low motivation [14]. Previous research has frequently reported low participation in postpartum lifestyle interventions, largely because women struggle to prioritize their own health while managing the demands of newborn care and household responsibilities [27–29]. The hybrid model, which combined in-person, synchronous virtual, and asynchronous video components, appeared to be particularly responsive to participants’ needs. Adherence to asynchronous pre-recorded sessions was highest, reflecting a preference for the flexibility these sessions provided. This is in line with findings from Barbosa et al. [30], who reported greater participation in telehealth-based interventions due to their adaptable format. Although adherence to telerehabilitation and in-person sessions was slightly lower, these rates remain favorable compared to similar interventions targeting postpartum populations [27, 28]. The intervention groups in those studies reported dropout rates of 6% and 31%, respectively, illustrating the challenges of sustaining engagement in this demographic. In contrast, the present study recorded no dropouts or adverse events in a 4-week intervention period, reinforcing the potential of hybrid and flexible delivery formats to enhance retention by accommodating the complex needs of postpartum women.

### Cardiovascular and functional outcomes

The physiological outcomes of this intervention indicate promising trends; however, given the small sample size and large variability, these findings should be interpreted with caution and warrant further investigation in larger-scale randomized control studies. The reduction in systolic BP, approaching the early pregnancy value, is particularly noteworthy. This change is consistent with findings from Hardy et al. [24], which emphasize that even small reductions in postpartum systolic BP can have substantial long-term effects on cardiovascular risk. Moreover, the 5-mmHg reduction exceeds the 2-mmHg MCID [24, 25], and the moderate ES [23] provides additional support for the cardiovascular benefits of the intervention. These results suggest that our designed postpartum rehabilitation program may have clinical relevance, potentially supporting cardiovascular health in postpartum women. In contrast, diastolic BP increased slightly by 1.3 mmHg. Although this change is minimal, it aligns with trends often observed in short-term interventions, which may not provide sufficient time to induce more substantial shifts in diastolic BP [31]. The weak ES further suggests that this increase is unlikely to have any clinical impact. This highlights the need for longer-duration interventions to potentially achieve more substantial reductions in diastolic BP.

Regarding functional capacity, the 6MWD improved by 12 meters. Even though this increase falls short of the 30-meter threshold typically considered clinically meaningful for functional capacity improvement [26], the moderate ES suggests a potential benefit from the intervention [23]. This finding is consistent with a previous pilot study, which reported similar improvements in aerobic capacity following short-term, moderate-intensity interventions in postpartum women [32]. Additionally, the moderate ES observed in the DASI, another measure of functional capacity, further supports the potential benefit of the intervention, suggesting that the rehabilitation program positively impacted participants’ functional performance and their perceived ability to perform daily activities.

In addition to systolic BP and 6MWD outcomes, the program also demonstrated favorable trends in postpartum anthropometrics. Participants showed improvements in body weight and BMI despite the short four-week duration and limited exclusive breastfeeding. The observed reductions in body weight and BMI, reaching first-trimester values, may be attributed to the nutritional education included in the intervention and align with previous evidence showing that combined exercise, dietary counseling, and breastfeeding can positively influence postpartum anthropometrics and metabolic recovery [33]. Breastfeeding, even when partial, is also known to support postpartum weight reduction and improve long-term cardiometabolic profiles, with particularly pronounced BP and cholesterol benefits for women with a history of HDP [34]. These findings suggest that integrating healthy eating during lactation and breastfeeding guidance within structured postpartum rehabilitation programs may further support favorable weight, BP, and cardiometabolic outcome trajectories in hypertensive postpartum women.

### Physical activity

Participants demonstrated notable increases in weekly moderate-intensity physical activity and daily step count, highlighting the effectiveness of postpartum rehabilitation program in promoting sustained physical activity, which is crucial for cardiovascular recovery and chronic disease prevention [35, 36]. Moreover, the observed reduction in sedentary time suggests a positive behavioral shift, which is known to have potential cardiovascular benefits for postpartum women, particularly those with a history of HDP [37].

The use of both self-reported physical activity data from the PPAQ and objective data from Fitbit devices adds robustness to the findings. Previous studies underscore that combining wearable technology with behavior change strategies, such as goal setting and health education, enhances participant engagement and supports long-term adherence to physical activity [38]. These results align with a recent review showing improved physical activity outcomes in postpartum women using digital tools [39].

### Quality-of-life and depression

Although the health-related quality of life domains did not show large changes, all of them demonstrated positive trends, suggesting the rehabilitation program positively impacted various aspects of well-being. The most noticeable improvement was in the SF-36 role-physical domain, which reflects an increase in participants’ ability to perform everyday physical tasks and manage physical demands [40]. This suggests that the program helped enhance participants’ physical functioning and confidence in handling day-to-day activities that require physical effort. Similar results were reported by Brites-Lagos et al., who found comparable benefits in postpartum women participating in a supervised postpartum exercise program [32].

In terms of depressive symptoms, both the PHQ-9 and EPDS showed a downward trend, indicating a reduction in depressive symptomatology. While the program did not specifically target mental health, qualitative feedback also revealed increased feelings of support, a greater sense of autonomy, and improved confidence. These psychosocial benefits reflect outcomes reported in similar postpartum group interventions, which emphasize the importance of support and engagement for emotional well-being [32, 41].

### Barriers, facilitators, and participant perspectives

Caregiving responsibilities and time constraints emerged as major barriers to participation. These challenges are consistent with previous studies, which have shown that postpartum women often face competing demands that limit their ability to engage in health interventions [27–29]. Following the intervention, additional barriers such as physical exertion and environmental distractions became more prominent. This shift underscores the evolving nature of challenges faced during the postpartum period and highlights the need for flexible and responsive intervention models that adapt as women transition from early recovery to more active caregiving and lifestyle roles [14].

Social support and access to professional guidance were identified as key facilitators of engagement and success in the program. Although group-based formats were generally well received, some participants expressed a preference for alternative formats, indicating that individual differences may influence the perceived value of group settings. These findings emphasize the importance of offering both in-person and virtual options to meet diverse needs. This consideration is especially important for women recovering from HDP, as they may place greater value on medically focused and structured approaches rather than recreational or socially oriented elements in their postpartum rehabilitation [42].

### Study limitations and future directions

Several limitations of this study should be acknowledged. First, the small sample size and limited recruitment may affect the interpretability and generalizability of findings. The absence of a control group also constrained the ability to make causal inferences, as the observed improvements may have reflected natural postpartum recovery rather than the effects of the intervention. In addition, the short program duration may have restricted the potential to observe meaningful long-term physiological or psychosocial outcome changes. Lastly, direct measures of cardiometabolic profiles were not conducted, limiting the interpretation of the program’s potential effects on cardiometabolic health. A future pilot study should refine the intervention, assess its feasibility in a larger and more diverse sample, and extend the program duration to enable a more comprehensive evaluation of outcomes. Following this, an efficacy trial using a randomized controlled design should be conducted to evaluate both the immediate and longer-term effects of the intervention. These trials should specifically focus on its impact on cardiovascular risk reduction, particularly in relation to improvements in vascular structure and function, cardiometabolic markers, along with other relevant health outcomes, to establish the intervention’s effectiveness and its potential for sustained benefits over time.

## Conclusion

This study suggests that a 4-week hybrid cardio-obstetrics rehabilitation program is feasible for postpartum women with a history of hypertensive pregnancy. Preliminary findings suggest potential benefits for BP, functional capacity, body weight and BMI, physical activity, and health-related quality of life. To establish the intervention’s efficacy and long-term impact, future research should incorporate extended program durations and well-designed randomized controlled trials with larger and more diverse samples.

## Supplementary information

Supplementary Information
