# Supplementary material for: Postpartum cardio-obstetrics rehabilitation program for women after hypertensive pregnancy: A single-arm proof-of-concept study
Source: Hypertens Res. 2026 Jan 28;49(4):1415–27. doi: 10.1038/s41440-026-02556-1 (PMC13050647; doi:10.1038/s41440-026-02556-1)
Supplement: Supplementary file 1 — Supplementary Information [file 41440_2026_2556_MOESM1_ESM.docx]

**Supplemental Table 1** Responses of the acceptability questionnaire

| Questions from questionnaire | Agree [n (%)] |
| --- | --- |
| Overall exercise program |  |
| The exercise program was engaging and useful | 5 (83.33) |
| The exercise program was beneficial for my health | 6 (100) |
| The exercise program provided helpful exercises I will continue using after it ended | 6 (100) |
| I enjoyed the combination of in-person, live Zoom, and pre-recorded sessions | 6 (100) |
| The combination of session types helped me stay active and engaged throughout the program | 5 (83.33) |
| Specific session |  |
| In-person |  |
| The format of session was easy to follow | 6 (100) |
| The format of session was demanding | 2 (33.33) |
| The format of session was time-consuming | 2 (33.33) |
| Virtual |  |
| The format of session was easy to follow | 6 (100) |
| The format of session was demanding | 2 (33.33) |
| The format of session was time-consuming | 2 (33.33) |
| Pre-recorded |  |
| The format of session was easy to follow | 6 (100) |
| The format of session was demanding | 2 (33.33) |
| The format of session was time-consuming | 2 (33.33) |

**Supplemental Table 2** Perceived benefits and barriers to physical activity before and after the rehabilitation program

|  | **Pre-training** | | | **Post-training** | | |
| --- | --- | --- | --- | --- | --- | --- |
|  | **Average score**  mean ± SD | **Agree**  n (%) | **Disagree**  n (%) | **Average score**  mean ± SD | **Agree**  n (%) | **Disagree**  n (%) |
| **Perceived Benefits Item** |  |  |  |  |  |  |
| Life enhancement subscale |  |  |  |  |  |  |
| My disposition is improved by exercise | 3.50 ± 0.55 | 6 (100.00) | 0 (0.00) | 3.50 ± 0.55 | 6 (100.00) | 0 (0.00) |
| Exercising helps me sleep better at night | 3.50 ± 0.84 | 5 (83.33) | 1 (16.67) | 3.17 ± 0.75 | 5 (83.33) | 1 (16.67) |
| Exercise helps me decrease fatigue | 2.67 ± 1.03 | 4 (66.67) | 2 (33.33) | 3.33 ± 0.82 | 5 (83.33) | 1 (16.67) |
| Exercising improves my self-concept | 3.00 ± 1.10 | 5 (83.33) | 1 (16.67) | 3.33 ± 0.82 | 5 (83.33) | 1 (16.67) |
| Exercising increases my mental alertness | 3.17 ± 0.75 | 5 (83.33) | 1 (16.67) | 3.17 ± 1.17 | 5 (83.33) | 1 (16.67) |
| Exercise allows me to carry out normal activities without becoming tired | 3.17 ± 0.75 | 5 (83.33) | 1 (16.67) | 3.17 ± 0.75 | 5 (83.33) | 1 (16.67) |
| Exercise improves the quality of my work | 3.33 ± 0.52 | 6 (100.00) | 0 (0.00) | 3.17 ± 1.17 | 5 (83.33) | 1 (16.67) |
| Exercise improves overall body functioning for me | 2.83 ± 1.17 | 3 (50.00) | 3 (50.00) | 3.50 ± 0.84 | 4 (66.67) | 2 (33.33) |
| Physical performance subscale |  |  |  |  |  |  |
| Exercise increases my muscle strength | 3.50 ± 0.55 | 6 (100.00) | 0 (0.00) | 3.67 ± 0.52 | 6 (100.00) | 0 (0.00) |
| Exercising increases my level of physical fitness | 3.50 ± 0.55 | 6 (100.00) | 0 (0.00) | 3.67 ± 0.52 | 6 (100.00) | 0 (0.00) |
| My muscle tone is improved with exercise | 3.00 ± 1.26 | 4 (66.67) | 2 (33.33) | 3.67 ± 0.52 | 6 (100.00) | 0 (0.00) |
| Exercising improves functioning of my cardiovascular system | 3.33 ± 0.82 | 5 (83.33) | 1 (16.67) | 3.83 ± 0.41 | 6 (100.00) | 0 (0.00) |
| Exercise increases my stamina | 3.33 ± 0.82 | 5 (83.33) | 1 (16.67) | 3.67 ± 0.52 | 6 (100.00) | 0 (0.00) |
| Exercise improves my flexibility | 3.17 ± 1.17 | 5 (83.33) | 1 (16.67) | 3.50 ± 0.55 | 6 (100.00) | 0 (0.00) |
| My physical endurance is improved by exercising | 3.17 ± 1.17 | 5 (83.33) | 1 (16.67) | 3.67 ± 0.52 | 6 (100.00) | 0 (0.00) |
| Exercise improves the way my body looks | 3.00 ± 0.63 | 5 (83.33) | 1 (16.67) | 3.50 ± 0.84 | 5 (83.33) | 1 (16.67) |
| Psychological outlook subscale |  |  |  |  |  |  |
| I enjoy exercise | 2.83 ± 0.98 | 5 (83.33) | 1 (16.67) | 3.50 ± 0.55 | 6 (100.00) | 0 (0.00) |
| Exercise decreases feelings of stress and tension for me | 3.00 ± 0.63 | 5 (83.33) | 1 (16.67) | 4.00 ± 0.00 | 6 (100.00) | 0 (0.00) |
| Exercise improves my mental health | 3.50 ± 0.55 | 6 (100.00) | 0 (0.00) | 4.00 ± 0.00 | 6 (100.00) | 0 (0.00) |
| Exercise gives me a sense of personal accomplishment | 3.67 ± 0.52 | 6 (100.00) | 0 (0.00) | 3.67 ± 0.52 | 6 (100.00) | 0 (0.00) |
| Exercising makes me feel relaxed | 3.33 ± 0.52 | 6 (100.00) | 0 (0.00) | 3.00 ± 1.10 | 5 (83.33) | 1 (16.67) |
| I have improved feelings of well-being from exercise | 3.33 ± 0.82 | 5 (83.33) | 1 (16.67) | 3.33 ± 0.82 | 5 (83.33) | 1 (16.67) |
| Social interaction subscale |  |  |  |  |  |  |
| Exercising lets me have contact with friends and persons I enjoy | 2.83 ± 0.98 | 5 (83.33) | 1 (16.67) | 3.00 ± 1.26 | 4 (66.67) | 2 (33.33) |
| Exercising is a good way for me to meet new people | 2.33 ± 0.82 | 3 (50.00) | 3 (50.00) | 3.17 ± 0.75 | 5 (83.33) | 1 (16.67) |
| Exercise is good entertainment for me | 3.00 ± 1.10 | 5 (83.33) | 1 (16.67) | 3.67 ± 0.52 | 6 (100.00) | 0 (0.00) |
| Exercising increases my acceptance by others | 3.00 ± 1.10 | 5 (83.33) | 1 (16.67) | 2.83 ± 1.17 | 4 (66.67) | 2 (33.33) |
| Preventative health subscale |  |  |  |  |  |  |
| I will prevent heart attacks by exercising | 3.17 ± 1.17 | 5 (83.33) | 1 (16.67) | 3.67 ± 0.52 | 6 (100.00) | 0 (0.00) |
| Exercising will keep me from having high blood pressure | 3.17 ± 0.75 | 5 (83.33) | 1 (16.67) | 3.50 ± 1.22 | 5 (83.33) | 1 (16.67) |
| I will live longer if I exercise | 3.67 ± 0.82 | 5 (83.33) | 1 (16.67) | 3.50 ± 0.84 | 5 (83.33) | 1 (16.67) |
| **Perceived Barriers Item** |  |  |  |  |  |  |
| Exercise environment subscale |  |  |  |  |  |  |
| Places for me to exercise are too far away | 3.00 ± 1.10 | 5 (83.33) | 1 (16.67) | 3.67 ± 0.52 | 6 (100.00) | 0 (0.00) |
| I am too embarrassed to exercise | 3.17 ± 1.17 | 5 (83.33) | 1 (16.67) | 3.67 ± 0.52 | 6 (100.00) | 0 (0.00) |
| It costs too much money to exercise | 3.67 ± 0.52 | 6 (100.00) | 0 (0.00) | 3.33 ± 0.82 | 5 (83.33) | 1 (16.67) |
| Exercise facilities do not have convenient schedules for me | 3.17 ± 0.75 | 5 (83.33) | 1 (16.67) | 3.50 ± 0.55 | 6 (100.00) | 0 (0.00) |
| I think people in exercise clothes look funny | 3.17 ± 1.33 | 4 (66.67) | 2 (33.33) | 3.67 ± 0.52 | 6 (100.00) | 0 (0.00) |
| There are too few places for me to exercise | 3.00 ± 1.10 | 5 (83.33) | 1 (16.67) | 3.67 ± 0.52 | 6 (100.00) | 0 (0.00) |
| Time expenditure subscale |  |  |  |  |  |  |
| Exercising takes too much of my time | 3.00 ± 0.00 | 6 (100.00) | 0 (0.00) | 3.33 ± 0.82 | 5 (83.33) | 1 (16.67) |
| Exercise takes too much time from family relationships | 3.00 ± 0.63 | 5 (83.33) | 1 (16.67) | 3.50 ± 0.55 | 6 (100.00) | 0 (0.00) |
| Exercise takes too much time from my family responsibilities | 3.17 ± 0.75 | 5 (83.33) | 1 (16.67) | 3.33 ± 0.82 | 5 (83.33) | 1 (16.67) |
| Physical exertion subscale |  |  |  |  |  |  |
| Exercise tires me | 2.67 ± 1.03 | 4 (66.67) | 2 (33.33) | 3.50 ± 0.55 | 6 (100.00) | 0 (0.00) |
| I am fatigued by exercise | 3.17 ± 0.75 | 5 (83.33) | 1 (16.67) | 3.50 ± 0.55 | 6 (100.00) | 0 (0.00) |
| Exercise is hard work for me | 2.50 ± 0.84 | 4 (66.67) | 2 (33.33) | 3.00 ± 0.89 | 4 (66.67) | 2 (33.33) |
| Family discouragement subscale |  |  |  |  |  |  |
| My spouse (or significant other) doesn’t encourage exercising | 3.50 ± 1.22 | 5 (83.33) | 1 (16.67) | 3.00 ± 1.10 | 5 (83.33) | 1 (16.67) |
| My family members do not encourage me to exercise | 3.50 ± 0.84 | 5 (83.33) | 1 (16.67) | 3.17 ± 1.17 | 5 (83.33) | 1 (16.67) |
| Assessed by a 4-point Likert Exercise Benefits and Barrier Scale (EBBS). | | | | | | |

**Supplemental Table 3** Participant perceptions of barriers and facilitators in cardiac rehabilitation

|  | **Average score**  mean ± SD | **Agree**  n (%) | **Disagree**  n (%) |
| --- | --- | --- | --- |
| **Barriers Item** |  |  |  |
| Health status perception |  |  |  |
| I didn't know about cardiac rehabilitation | 1.33 ± 0.52 | 0 (0.00) | 6 (100.00) |
| I don't need cardiac rehabilitation | 1.50 ± 0.55 | 0 (0.00) | 6 (100.00) |
| I already exercise at home or in my community | 2.33 ± 1.75 | 2 (33.33) | 4 (66.67) |
| My doctor didn't feel it was necessary | 1.33 ± 0.52 | 0 (0.00) | 6 (100.00) |
| Many people with heart problems don't go, and they are fine | 1.33 ± 0.52 | 0 (0.00) | 6 (100.00) |
| I can manage my heart problem on my own | 1.67 ± 0.82 | 0 (0.00) | 6 (100.00) |
| I think I was referred, but the rehab program didn't contact me | 1.17 ± 0.41 | 0 (0.00) | 6 (100.00) |
| It took too long to get referred and into the program | 1.33 ± 0.52 | 0 (0.00) | 6 (100.00) |
| I prefer to take care of my health alone, not in a group | 2.00 ± 1.10 | 1 (16.67) | 5 (83.33) |
| Logistic factors |  |  |  |
| Distance | 1.33 ± 0.52 | 0 (0.00) | 6 (100.00) |
| Cost | 1.83 ± 1.17 | 1 (16.67) | 5 (83.33) |
| Transportation | 1.67 ± 1.21 | 1 (16.67) | 5 (83.33) |
| Family responsibilities | 2.83 ± 1.72 | 3 (50.00) | 3 (50.00) |
| Severe weather | 1.33 ± 0.52 | 0 (0.00) | 6 (100.00) |
| Travel | 1.33 ± 0.52 | 0 (0.00) | 6 (100.00) |
| Work/time conflicts |  |  |  |
| Time constraints | 3.17 ± 0.75 | 2 (33.33) | 4 (66.67) |
| Work responsibilities | 2.00 ± 1.10 | 1 (16.67) | 5 (83.33) |
| Functional status |  |  |  |
| I find exercise tiring and/or painful | 1.83 ± 1.17 | 1 (16.67) | 5 (83.33) |
| I don't have energy | 1.50 ± 0.55 | 0 (0.00) | 6 (100.00) |
| Other health problems prevent me from going | 1.83 ± 0.75 | 0 (0.00) | 6 (100.00) |
| I am too old | 1.50 ± 0.55 | 0 (0.00) | 6 (100.00) |
| **Facilitators Item** |  |  |  |
| Accessibility |  |  |  |
| Having the cardiac rehabilitation program closer to my home | 4.33 ± 0.82 | 5 (83.33) | 1 (16.67) |
| Receiving the sessions in tele-health/web-based format | 3.67 ± 1.86 | 5 (83.33) | 1 (16.67) |
| Receiving financial aid to cover the transport fees | 3.83 ± 1.60 | 4 (66.67) | 2 (33.33) |
| Support |  |  |  |
| Receiving encouragement from hospital nurse/physician to attend this program | 3.67 ± 1.97 | 4 (66.67) | 2 (33.33) |
| Motivation |  |  |  |
| Starting the program within 2 weeks after my discharge from the hospital | 2.67 ± 1.51 | 3 (50.00) | 3 (50.00) |
| Receiving rewards for completing a given number of sessions | 2.83 ± 1.60 | 2 (33.33) | 4 (66.67) |
| Program Content |  |  |  |
| Receiving nutritional assessment and "how-to-cook" classes | 3.83 ± 1.94 | 5 (83.33) | 1 (16.67) |
| Receiving psychological assessment and specific counselling | 3.33 ± 1.97 | 3 (50.00) | 3 (50.00) |
| Receiving stress management classes | 3.83 ± 1.60 | 4 (66.67) | 2 (33.33) |
| Having received dance classes as exercise interventions | 2.33 ± 1.63 | 1 (16.67) | 5 (83.33) |
| Assessed by a 5-point Likert Cardiac Rehabilitation Barriers Scale (CRBS) and Cardiac Rehabilitation Facilitators Scale (CRFS). | | | |

(a) **
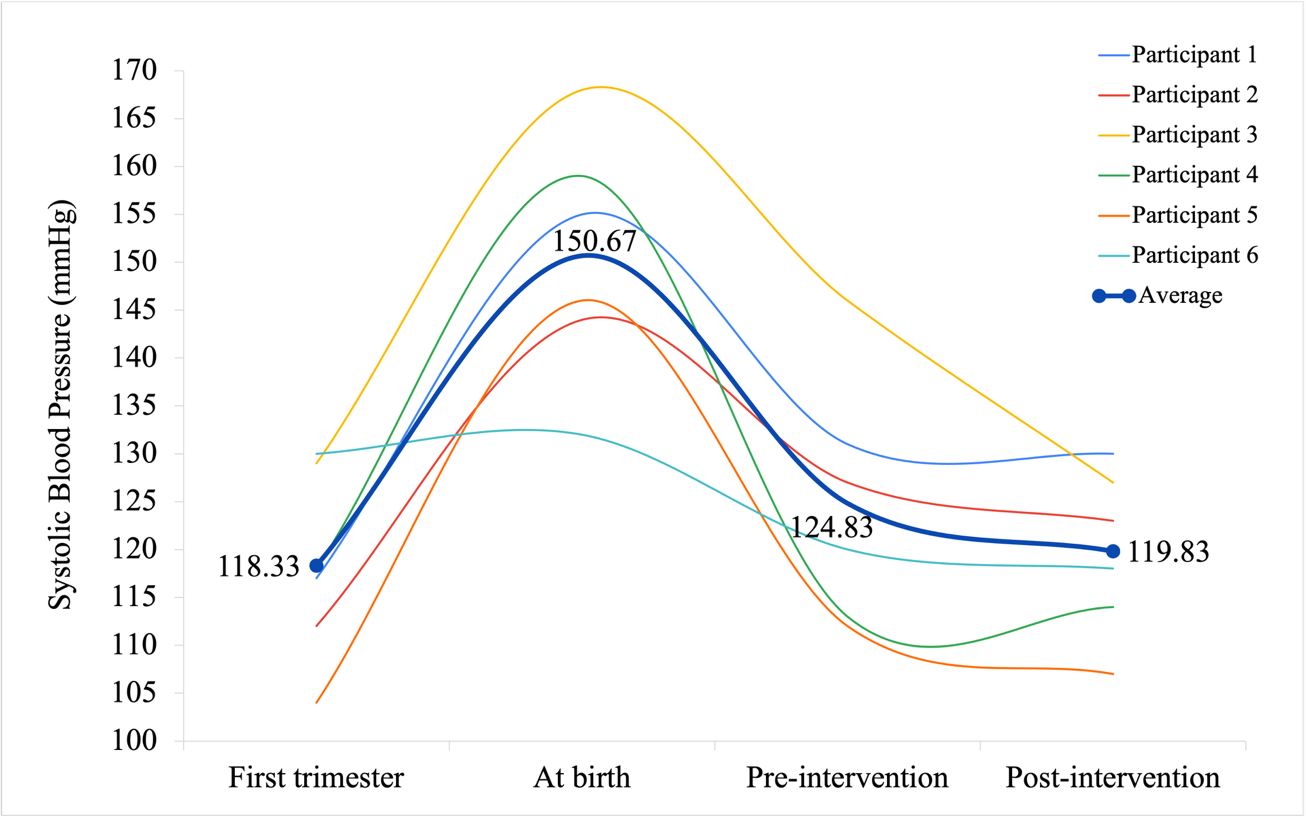
**

(b) **
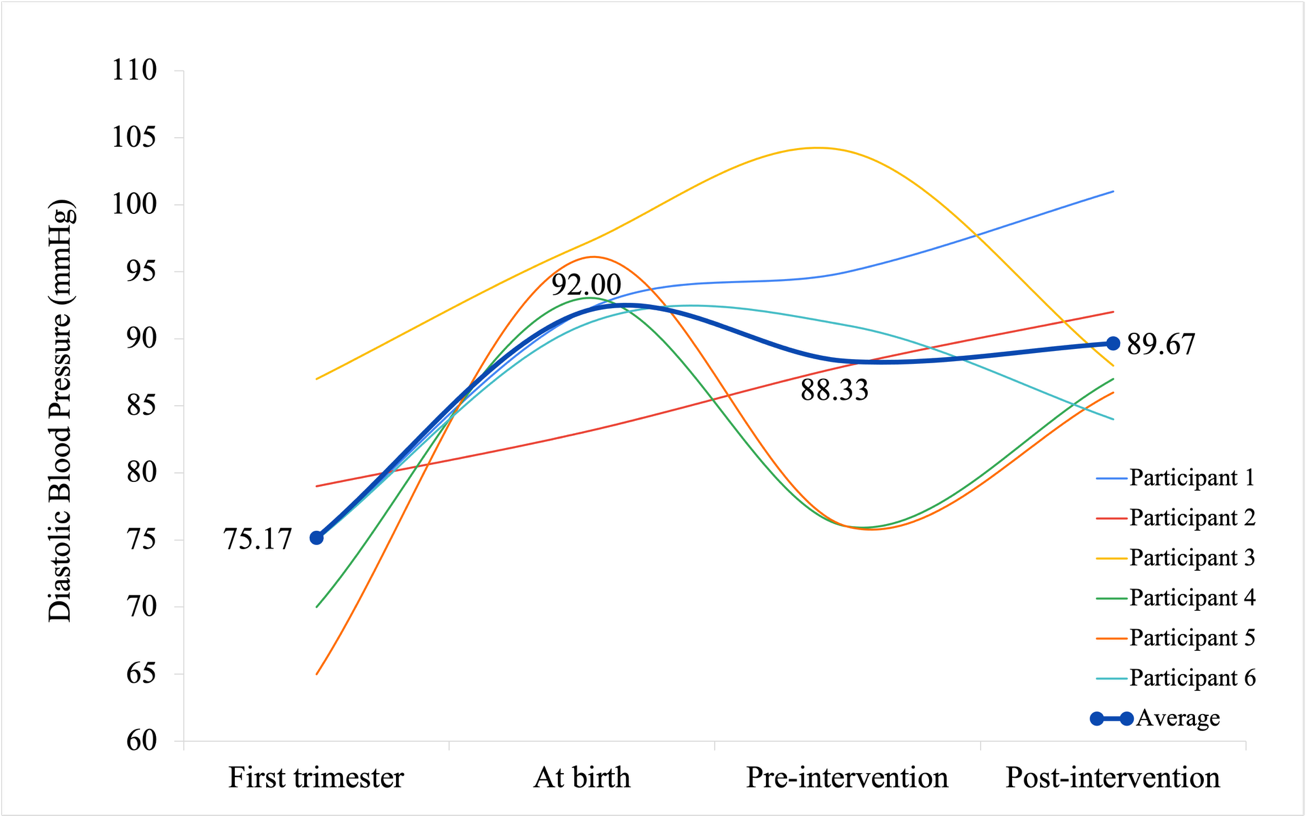
**

**Supplemental Figure 1** Individual trajectories of (a) systolic and (b) diastolic blood pressure
